# Mycoplasma Genitalium Among Women With Nongonococcal, Nonchlamydial Pelvic Inflammatory Disease

**DOI:** 10.1155/IDOG/2006/30184

**Published:** 2006-05-16

**Authors:** Catherine L. Haggerty, Patricia A. Totten, Sabina G. Astete, Roberta B. Ness

**Affiliations:** ^1^University of Pittsburgh, Pittsburgh, PA 15260, USA; ^2^Department of Epidemiology, University of Pittsburgh, 130 DeSoto Street, 516B Parran Hall Pittsburgh, PA 15261, USA; ^3^Department of Medicine, Division of Infectious Diseases, University of Washington, Seattle, WA, USA

## Abstract

Pelvic inflammatory disease (PID) is a frequent condition of young women, often resulting in reproductive morbidity. Although *Neisseria gonorrhoeae* and/or *Chlamydia trachomatis* are/is recovered from approximately a third to a half of women with PID, the etiologic agent is often unidentified. We need PCR to test for *M genitalium* among a pilot sample of 50 women with nongonococcal, nonchlamydial endometritis enrolled in the PID evaluation and clinical health (PEACH) study. All participants had pelvic pain, pelvic organ tenderness, and leukorrhea, mucopurulent cervicitis, or untreated cervicitis. Endometritis was defined as ≥5 surface epithelium neutrophils per ×400 field absent of menstrual endometrium and/or ≥2 stromal plasma cells per ×120 field. We detected *M genitalium* in 7 (14%) of the women tested: 6 (12%) in cervical specimens and 4 (8%) in endometrial specimens. We conclude that *M genitalium* is prevalent in the endometrium of women with nongonococcal, nonchlamydial PID.

## INTRODUCTION

*Mycoplasma genitalium* was first identified in the early 1980s among men with nongonococcal urethritis [1]. *M genitalium* is extremely difficult to culture, but the use of polymerase chain reaction (PCR) technology has allowed research into the pathogenicity of this organism. Numerous studies have confirmed the role of *M genitalium* in drug resistant nongonococcal urethritis [2–18], and four relatively recent studies have shown *M genitalium* to be associated with cervicits [19–22], independent of *Chlamydia trachomatis* and *Neisseria gonorrhoeae* [20–22]. Few studies have examined *M genitalium* among women with pelvic inflammatory disease (PID), the common infection and inflammation of the female upper genital tract which may lead to major reproductive morbidity including infertility, chronic pelvic pain, ectopic pregnancy, and recurrent PID [23]. Whereas serological studies of *M genitalium* and PID among women have been suggestive but inconclusive [24, 25], PCR studies have associated *M genitalium* with clinically suspected PID [19–26] and acute endometritis [27]. Only one of these studies confirmed the diagnosis of PID histologically [27], and no studies have examined *M genitalium* among a population of US women with PID.

*C trachomatis* and/or *N gonorrhoeae* account for approximately a third to half of PID cases [30, 32–36, 38–50]. Thus, since up to 70% of PID cases have a largely unidentified etiology, the discovery of new pathogens associated with upper genital tract infection in women would be informative. Anaerobic gram-negative rods and bacterial vaginosis have been associated with PID [30–32, 36–38, 43, 45–50], and we have recently shown this association to be independent of gonococcal and chlamydial infection [51]. It is feasible that *M genitalium* may also be an etiologic agent in nongonococcal, nonchlamydial PID, as it has been found to induce salpingitis experimentally in monkeys [52, 53], has been shown to adhere to human fallopian tube epithelial cells in organ culture [54], and has been detected in fallopian tube tissue in a woman with salpingitis [55]. Further, *M genitalium* has been shown to adhere to human spermatozoa, and therefore may potentially be carried by motile sperm to the female upper genital tract [56].

## SUBJECTS

We conducted a pilot substudy to determine the prevalence of *M genitalium* among a sample of urban US women with nongonococcal, nonchlamydial PID using specimens collected as part of the PID evaluation and clinical health (PEACH) study. The methods of PEACH study participant recruitment, data collection, and followup have been described in detail elsewhere [57]. Briefly, women aged 14 to 37 years were recruited from emergency departments, OB/GYN clinics, STD clinics, and private practices at thirteen clinical sites located throughout the eastern, southern, and central regions of the US between March 1996 and February 1999. Women with clinically suspected PID who gave informed consent were eligible for the PEACH study. All participants had clinically suspected PID: pelvic, pain, pelvic organ tenderness; and leukorrhea, mucopurulent cervicitis, or untreated cervicitis. Eight hundred thirty one women met all of the inclusion criteria and were enrolled into the PEACH study. Six hundred fifty four women consented to endometrial biopsies. Histologic evidence of endometritis, defined as ≥ 5 surface epithelium neutrophils per ×400 field absent of menstrual endometrium and/or ≥ 2 stromal plasma cells per ×120 field, was found among 311 women.

## METHODS

Cervical and endometrial specimens from a sample of 50 women with nongonococcal, nonchlamydial endometritis were tested for *M genitalium* using the MgPa-IMW PCR assay targeting the MgPa gene [19]. For all samples testing positive for *M genitalium*, a second MgPa PCR assay was performed using another aliquot of the sample to rule out PCR product contamination or cross-contamination; all samples initially positive were verified as positive in this confirmatory test. Frequencies and Pearson's correlations were calculated using SAS version 8.2 for Windows.

## RESULTS

We detected *M genitalium* in 7 (14%) of the women tested. Twelve percent of cervical specimens and 8 percent of endometrial specimens were positive for *M genitalium*, and infection at these sites was highly correlated (Pearson's correlation 0.57, *p* = 0.0001), with 75% of endometrial positive cases also positive at the cervix and 50% of cervical positive cases also positive in the endometrium. Both plasma cells and neutrophils were identified in 3 (75%) of the 4 women with endometrial *M genitalium* compared to 18 (42%) of 43 women without *M genitalium* in the endometrium, although these findings were not statistically significant.

## DISCUSSION

We identified *M genitalium* frequently among an urban US population of women with nongonococcal, nonchlamydial PID. The overall prevalence of 14% is similar to that found in prior PCR investigations among Kenyan women with histologically confirmed endometritis (16%) [27] and United Kingdom women with clinically suspected PID (13%) [26]. In the PEACH study, about 14% of women were infected with *C trachomatis*, 15% were infected with *N gonorrhoeae*, and 5% were coinfected [58]. Thus, *M genitalium* was as prevalent as *C trachomatis* or *N gonorrhoeae* among this population of women with PID.

Most women with PID are treated with antibiotics directed toward *N gonorrhoeae* and/or *C trachomatis*, despite the fact that these bacterial pathogens account for only a third to half of PID cases. Indeed, in the PEACH study the majority of women, approximately 60%, had nongonococcal, nonchlamydial PID [58]. Over a third of the women in the PEACH study had persistent endometritis at 30 days [58] and over a third experienced chronic pelvic pain subsequent to baseline [59], suggesting that a sizeable portion of women in the PEACH study had ongoing inflammation and infection. Women in the PEACH study were treated with a combination of cefoxitin and doxycycline [58], a PID treatment regimen currently recommended by the centers for disease control and prevention. No studies have examined the efficacy of this or any other treatment regimen among women with *M genitalium* PID. However, several attributes of *M genitalium* suggest it is resistant to cefoxitin and doxycycline. First, mycoplasmal bacteria lack a cell wall, and are thus resistant to cell wall inhibiting antibiotics, including penicillin and cephalosporin. Second, *M genitalium* has been found to persist among men treated with tetracyclines for nongonococcal urethritis [60]. Antibiotic resistance among *M genitalium* strains may lead to persistent or recurrent infection among women with PID, resulting in chronic inflammation and infection of the lower and upper genital tract. Further studies are needed to determine the efficacy of PID treatment regimens for the eradication of endometrial and tubal *M genitalium*.

Successful treatment of PID is critical for the prevention of subsequent reproductive morbidity. We have previously shown that women in the PEACH study with *N gonorrhoeae* or *C trachomatis* identified in the endometrium were not more likely to experience infertility, chronic pelvic pain, or recurrent PID than those negative for each [59]. Further, those who tested positive for nongonococcal bacteria were generally more likely to experience reproductive morbidity than were women with endometrial gonococcal infection (infertility rates were 13% for *N gonorrhoeae*, 19% for *C trachomatis*, 22% for anaerobic bacteria, 27% for *U urealyticum*, and 17% for *M hominis*; chronic pelvic pain rates were 27% for *N gonorrhoeae*, 21% for *C trachomatis*, 33% for anaerobic bacteria, 41% for *U urealyticum*, and 54% for *M hominis*). Thus, women with nongonococcal PID may be at greater risk for adverse outcomes. Few studies have examined reproductive sequelae attributed to *M genitalium* upper genital tract infection, but *M genitalium* antibodies have been identified more frequently (22% versus 6%) among women with tubal factor infertility compared to women with nontubal factor infertility [61].

Given the frequency of *M genitalium* among women with PID demonstrated in this report and in those by previous investigators [19, 26, 27], we recommend that treatment for PID be reevaluated for its effectiveness against nongonococcal, nonchlamydial PID. *M genitalium* has demonstrated susceptibility to macrolides, with azithromycin being the most active, and variable resistance to fluroquinolones, including ciprofloxacin [60, 62]. A newer quinolone, moxiflocacin, has recently been shown to exhibit better activity against *M genitalium* [63]. Further study of alternative regimens for the treatment of *M genitalium* PID and the prevention of adverse reproductive sequelae is needed.
